# Evolocumab early reduces IL-1β/IL-17A and improves left ventricular function in STEMI: an observational study in the real world

**DOI:** 10.3389/fphar.2026.1800539

**Published:** 2026-05-08

**Authors:** Menglong Zhang, Jingyu Liu, Jingxian Wang, Anran Jing, Junfeng Zhang, Changping Li, Zhuang Cui, Yin Liu, Jing Gao

**Affiliations:** 1 Thoracic Clinical College, Tianjin Medical University, Tianjin, China; 2 Department of Cardiology, Tianjin Chest Hospital, Tianjin, China; 3 Department of Cardiology, Yantai Yuhuangding Hospital, Yantai, China; 4 School of Public Health, Tianjin Medical University, Tianjin, China; 5 Cardiovascular Institute, Tianjin Chest Hospital, Tianjin, China; 6 Tianjin Chest Hospital, Tianjin University, Tianjin, China; 7 Tianjin Key Laboratory of Cardiovascular Emergency and Critical Care, Tianjin, China

**Keywords:** evolocumab, inflammatory factors, left ventricular function, real world study, ST-elevation-myocardial infarction

## Abstract

**Background:**

While proprotein convertase subtilisin/kexin type 9 inhibitors (PCSK9i) effectively lower low-density lipoprotein cholesterol (LDL-C) levels and reduce the incidence of major adverse cardiovascular events (MACE) in patients with ST-segment elevation myocardial infarction (STEMI), the anti-inflammatory effects and impact on early cardiac remodeling of PCSK9i remain unclear. This study investigated the effects of PCSK9i on early inflammatory factors [interleukin (IL)- 1β, IL-18, IL-17A, and C-C chemokine receptor 2 (CCR2)] and left ventricular (LV) function in STEMI patients.

**Methods:**

Totally 257 STEMI participants were divided into 2 groups based on their real-world prescriptions: those treated with statin only (statin group) and those treated with statin in addition to the evolocumab (evolocumab group). All patients were observed for 12 weeks.

**Results:**

At 4 weeks, the evolocumab group exhibited a greater reduction in IL-1β compared to statin alone (−29.35% vs −25.27%, *P* = 0.012). Similarly, IL-17A decreased more significantly with evolocumab than with statin therapy (−30.22% vs −25.35%, *P* = 0.023). By 12 weeks, evolocumab significantly improved left ventricular ejection fraction (LVEF) especially in the patients with LVEF<50% at baseline (11.36% vs 7.40%, *P* = 0.014) and increased the incidence of LV global function improvement (ΔEF≥ 5%: 78.2% vs 55.1%, *P* < 0.001) compared to statin alone. Multivariate analysis identified the use of evolocumab, reductions in IL-1β and IL-17A at 4 weeks as independent predictors of ΔEF≥ 5% at 12 weeks. Mediation analysis showed that 8.74% and 9.60% of LV functional improvement were attributable to reductions in IL-1β and IL-17A respectively.

**Conclusion:**

Our findings support evolocumab’s role beyond lipid-lowering, suggesting that early PCSK9i as a potential strategy to mitigate early inflammation-driven cardiac dysfunction in STEMI management.

## 1 Introduction

Despite optimized secondary prevention after percutaneous coronary intervention (PCI), patients with ST-segment elevation myocardial infarction (STEMI) remain at high risk for recurrent major adverse cardiovascular events (MACE). High-intensity statin therapy is recommended to lower low-density lipoprotein cholesterol (LDL-C) for acute coronary syndrome (ACS) patients who are not contraindicated by both the European Society of Cardiology (ESC) and American Heart Association/American College of Cardiology (AHA/ACC) guidelines (McCune et al., 2015). In addition to traditional risk factors, other drivers of residual risk are now considered to be significantly associated with the occurrence of MACE (Cannon et al., 2004; Dhindsa et al., 2020; Jernberg et al., 2015; Sabatine et al., 2017), among which inflammation plays a pivotal role in atherosclerosis progression and plaque vulnerability (Di Muro et al., 2025). In addition, necrotic cardiomyocytes after STEMI induce transient cytokine storms (He et al., 2022). The severity of inflammation after STEMI serves as a primary determinant of cardiac remodeling and function, and dysregulation or prolongation of inflammation can lead to adverse cardiac remodeling and MACE (Mahtta et al., 2020; Wang et al., 2018). Epidemiological studies have reported that the incidence of heart failure after STEMI is approximately 25% and about 40% of myocardial infarction (MI) is accompanied by left ventricular (LV) systolic dysfunction (Albert and Lewis, 2008). Despite PCI intervention, a substantial proportion (30%–35%) of STEMI patients experience cardiac remodeling, leading to increased adverse outcomes and mortality after STEMI (Karabağ et al., 2019; Ola et al., 2018). Therefore, management of inflammation after STEMI is critical. So far, the anti-inflammatory effect of statins in patients with cardiovascular diseases has been confirmed in multiple clinical studies (Albert et al., 2001; Golia et al., 2014; Nissen et al., 2004; Satny et al., 2021), while other cytokine-targeted therapies, such as canakinumab and tocilizumab, remain in experimental phases, demanding rigorous assessment of their therapeutic potential and safety concerns (Holte et al., 2017; Mahtta et al., 2020). The current challenge focuses on developing innovative inflammation-modulating therapies that can effectively enhance cardiac function in STEMI patients while demonstrating clinical safety.

Achieving intensive lipid lowering can be facilitated by proprotein convertase subtilisin/kexin type 9 (PCSK9) inhibition (Savage et al., 2025). The effects of PCSK9 inhibitors (PCSK9i) in reducing LDL-C levels, improving lipid metabolism, and reducing long-term MACE in patients with STEMI have been verified in multiple large-scale clinical trials (Dhindsa et al., 2020; Mahtta et al., 2020; Steg et al., 2019). However, whether PCSK9i exert comparable anti-inflammatory effects to statins remains controversial in current evidence. Two meta-analyses published in 2016 and 2018 and several original studies demonstrated that PCSK9i significantly reduced LDL-C with neutral effects on hypersensitive C-reactive protein (hsCRP) levels (Liu and Frostegård, 2018; Sahebkar et al., 2016). However, several RCTs have reported more significant reductions in hsCRP levels and macrophage activity in PCSK9i-treated groups compared to controls. A few studies also showed that PCSK9i can rapidly reduce inflammatory factor levels in patients with STEMI after PCI (Cao et al., 2018; Ji et al., 2023; Ou et al., 2022). These findings primarily derive from small-scale studies with limited sample sizes, underscoring the need for more large-scale trials to validate anti-inflammatory effects of PCSK9i.

Interleukin-17A (IL-17A) is produced by γδT cell in STEMI, which is a cytokine with a potential role in STEMI. IL-17A plays an important role in the immune response and affects the production of different inflammatory mediators in several types of cells, involved in the damage or scar process in myocardial tissue. A study demonstrates that a deficiency in IL-17A or γδT cells improved survival after 7 days, limiting infarct expansion and fibrosis in non-infarcted myocardium and alleviating LV dilatation and systolic dysfunction after 28 days of MI (Ji et al., 2023). Some studies have shown that PCSK9 can change T cell programming and transform into γδT cells by affecting the production of modified LDL-C in hepatocytes (Liu and Frostegård, 2018; Yan et al., 2012).

MI causes cell death and tissue necrosis and is associated with infiltration of inflammatory cells, primarily neutrophils and monocytes/macrophages, into the infarct area. Early after the initial occlusion, monocytes penetrate into the infarct area and differentiate into macrophages that display inflammation (M1 macrophages). C-C motif ligand 2 (CCL2) released by M1 macrophages leads to enhanced recruitment of further circulating monocytes from the circulation. CCL2 and its receptor, chemokine C-C motif receptor 2 (CCR2), are crucial in the recruitment and migration of M1 macrophages to the infarct area (Weipert et al., 2025), which perpetuates inflammation at the injured site. Over time, this persistent inflammation can lead to unfavorable tissue remodeling and impair heart function (Wen et al., 2026). Macrophages can produce large amounts of interleukin-1β (IL-1β) and interleukin-18 (IL-18) in response to the NOD-like receptor thermal protein domain associated protein 3 (NLRP3) inflammatory body activator. Activation of the NLRP3 inflammatory body drives the release of IL-1β in cardiac fibroblasts and the pyroptosis of cardiomyocytes promoted by IL-18, thereby promoting cardiac inflammation and remodeling in MI (Li et al., 2023; Takahashi, 2019). A study showed that CCL2/CCR2 signaling promotes and amplifies inflammatory damage mediated and amplified by NLRP3/cysteine-aspartic acid protease 1 (Caspase-1)/IL-1β. Knocking out the CCR2 gene can effectively reduce myocardial infarction size and downregulate inflammatory mediators and NLRP3/Caspase-1/IL-1β signaling. After knocking out or inhibiting the CCR2 gene, transforming growth factor-β (TGF-β) levels were significantly reduced, the level of myocardial fibrosis was significantly reduced, and cardiac function was protected (Shen et al., 2024). Studies in mice have shown that the application of CCR2 inhibitors improves left ventricular ejection fraction (LVEF) and LV wall thickening after myocardial infarction, reduces collagen scar formation, and reduces selectively activated macrophages in the infarct area (Weipert et al., 2025). The transition from acute injury to chronic fibrosis after MI in mice is mediated by CCL2/CCR2 signaling in macrophages through the NLRP3 inflammatory cascade and phenotypic switching.

There is evidence that PCSK9i reduced monocyte CCR2 expression, which is probably related to lipids and inflammation-immune (Wang et al., 2023). In addition, PCSK9 can directly activate the NLRP3 inflammasome and promote the secretion of and IL-18 and IL-1β, which promotes the production of IL-17A (Kim et al., 2019). These studies suggest that PCSK9i may have beneficial effects on early LV function in STEMI patients through inflammation-immune mechanisms.

Whether PCSK9i have pleiotropic effects in STEMI patients remains to be further explored. To demonstrate the inflammation-immune mechanism of PCSK9i, we designed this single-center, prospective and non-interventional Real-World study. The primary purpose of this study was to investigate the effects of evolocumab treatment on early changes in inflammatory factors (after 1 and 4 weeks of treatment) in patients with STEMI. Our secondary purpose of this study was to explore the impact of evolocumab treatment on early left ventricular function (after 4 and 12 weeks of treatment) in patients with STEMI.

## 2 Materials and methods

### 2.1 Study design and population

An observational single-center cohort study was conducted on totally 257 patients with STEMI at Tianjin Chest Hospital, China between September 2020 and March 2022. STEMI was confirmed by coronary angiography, electrocardiogram and myocardial injury biomarkers. The patients were divided into 2 groups based on their real-world prescriptions in this observational study: those treated with statin only (statin group, N = 138) and those treated with statin in addition to the evolocumab (evolocumab group, N = 119). All patients with STEMI are treated with moderate-intensity statins, rosuvastatin (10 mg) or atorvastatin (20 mg) daily throughout the study period. Patients in evolocumab group received evolocumab treatment within 72h immediately after diagnosis of STEMI, with subcutaneous administration of 140 mg evolocumab once every 2 weeks. After discharge, all patients were seen as outpatients on week 4 and 12. Primary endpoints included changes in inflammatory markers after 4 weeks and changes in LV functional parameters after 12 weeks.

**FIGURE 1:**
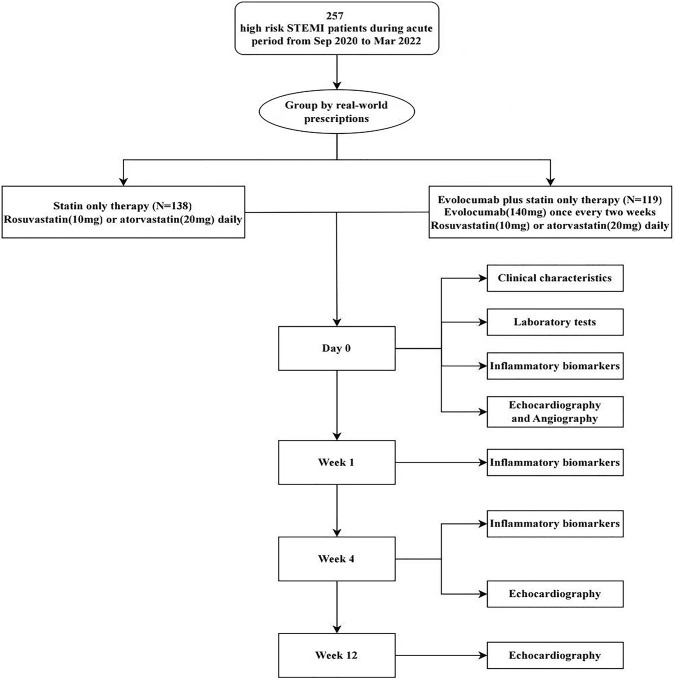
Study flowchart.

The study protocol has been reviewed and approved by the Ethics Committee (IEC) of Tianjin Chest Hospital, China (No: 2019KY-019-01). Written informed consent was obtained from all participants before enrollment. The trial conformed to the principles outlined in the Declaration of Helsinki. All patients were followed up for 12 weeks (Figure 1).

### 2.2 Inclusion and exclusion criteria

The recruitment process is as follows: (1) for patients who were received the lipid lowering therapy, assess whether they are suitable for the trial according to the inclusion and exclusion criteria; (2) for patients who fulfill the trial criteria, introduce and explain the study; patients will be enrolled only after they agree to participate and provide signed informed consent; (3) for patients who refuse to sign the informed consent form, withdraw them from this trial, and perform conventional clinical practice.

Inclusion criteria: (1) Patients aged ≥18 and ≤85 with recent hospitalization for acute phase ACS; (2) Prior to inclusion in the study, patients who received intensive statins for more than 4 weeks (the same dose of statin therapy has been sustained for the past 4 weeks), having LDL-C levels ≥70 mg/dl (≥1.8 mmol/L) or non-HDL-C ≥100 mg/dl (≥2.6 mmol/L); (3) Prior to inclusion in the study, patients who received moderate-intensity statin therapy for more than 4 weeks (the same dose of statin therapy has been sustained for the past 4 weeks), having LDL-C levels ≥90 mg/dl (≥2.3 mmol/L) or non-HDL-C ≥120 mg/dl (≥3.1 mmol/L); (4) Prior to inclusion in the study, patients who do not receive any statin therapy or discontinue statin therapy, having LDL-C ≥125 mg/dl (≥3.2 mmol/L) or non-HDL-C ≥155 mg/dl (≥4.0 mmol/L); and (5) Prior to inclusion in the study, patients who do not receive any statin therapy or discontinue statin therapy, having LDL-C ≤125 mg/dL (≤3.2 mmol/L).

Exclusion criteria: (1) Patients with clinically unstable conditions (e.g., hemodynamic or electrocardiographic instability); (2) Patients with estimated glomerular filtration rate (eGFR) < 30 mL/min/1.73 m^2^ or aspartate aminotransferase (AST)/alanine aminotransferase (ALT) levels >3 times the upper limit of normal; (3) Patients with known hypersensitivity to any study-related medications; (4) Patients who had received lipid-lowering therapy other than statin treatment; (5) Patients with autoimmune diseases (e.g., Crohn’s disease, systemic lupus erythematosus), recent infectious diseases, or those receiving immunosuppressive therapy (e.g., oral corticosteroids, nonsteroidal anti-inflammatory drugs, or cyclosporine).

### 2.3 Baseline clinical data and laboratory measurements

Baseline clinical data were assessed at admission, including: (1) Demographic characteristics (sex, date of birth), (2) Physical condition (height, weight, temperature, blood pressure, heart rate), (3) Past medical history [hypertension, diabetes, MI, PCI, coronary artery bypass grafting (CABG)], (4) Treatment history (coronary heart disease, name of medication used), (5) Family history (hypertension, diabetes, coronary heart disease, hyperlipidemia, stroke), (6) Lifestyle (smoking, alcohol consumption), (7) Current medical history.

Laboratory tests include: blood routine, blood glucose, liver function test (albumin, total bilirubin and direct bilirubin, alkaline phosphatase, aspartate aminotransferase, alanine aminotransferase), renal function test (serum creatinine, blood urea nitrogen, estimated glomerular filtration rate); lipid profile [LDL-C, high-density lipoprotein cholesterol (HDL-C), total cholesterol (TC), etc.]; myocardial injury biomarkers [serum creatine kinase (CK), creatine kinase isoenzyme (CK-MB), cardiac troponin I (TnI), and cardiac troponin T (TnT)], echocardiography [left atrial diameter (LAD), left ventricular end-diastolic diameter (LVDd), LVEF)] and other laboratory parameters such as hsCRP and N-terminal pro-B-type natriuretic peptide (NT-proBNP). All baseline laboratory tests were measured using standard clinical laboratory techniques blood samples were taken after an overnight fasting (≥8 h) on the day of admission.

### 2.4 Measurement of inflammatory biomarkers

Blood samples were collected in tubes with EDTA (ethylene diamine tetra-acetic acid) anticoagulant after an overnight fast (≥8 h) in the morning and centrifuged at 3000 rpm at 4 °C for 10 min to obtain serum at admission, 1 week, and 4 weeks post-treatment. All samples were stored at −80 °C in the Biobank of Tianjin City Chest Hospital until analysis. Serum IL-1β, IL-18, IL-17A, and CCR2 levels were measured by enzyme-linked immunosorbent assay (ELISA) kit (R& D Systems, Quanzhou, Fujian, CHN).

### 2.5 Coronary angiography and PCI

All patients underwent coronary angiography (CAG) and PCI upon admission to the hospital. CAG and PCI were performed by two qualified and experienced interventional cardiologists. The radial or femoral artery was punctured using the Seldinger puncture method to fully expose all segments of the coronary artery, and other positions were added if necessary. Two experts jointly assessed ischemia related artery and stenosis degree. Stents were selected according to the degree of coronary stenosis. The time from admission to balloon expansion and the number of stents implanted were recorded before and after operation. All patients received subcutaneous injection of low molecular weight heparin every 12 h for 1 week and treated with aspirin 100 mg and clopidogrel 75 mg daily after PCI.

### 2.6 Echocardiography

All patients underwent echocardiography at admission, 4 weeks and 12 weeks. Echocardiography was performed only by strictly trained attending echocardiographers or under their direct supervision in clinically stable patients, in accordance with current European guidelines to ensure high-standard echocardiographic examinations. All echocardiographers were blinded to patient allocation and final study analysis, and all LVEF measurements were performed offline by two independent sonographers who had no access to clinical or laboratory data, including group data. Left ventricular functional parameters including LVEF, LVDd, and LAD were calculated on dedicated workstations. LVEF was routinely measured by the modified Simpson’s rule method.

Definition of left ventricular global function improvement: An increase in LVEF after treatment compared with that before treatment greater than 5% (denoted as ΔEF≥ 5%) (Bax et al., 1999).

### 2.7 Statistical analysis

The Kolmogorov-Smirnov test was used to assess the distribution of variables. Continuous variables with normal or near-normal distributions were expressed as mean ± standard deviation (SD), while non-normally distributed data were presented as median with interquartile range (25th percentile, 75th percentile). Categorical variables were reported as numbers and percentages, and compared using the chi-square test or Fisher’s exact test as appropriate. The student's t-test or Mann-Whitney U test was used to compare baseline characteristics and levels and changes in observed parameters between the two groups as appropriate.

Generalized estimating equations (GEE) with an identity link function and Gaussian family were used to evaluate the effect of evolocumab on inflammatory biomarkers (IL-1β, IL-18, IL-17A, CCR2, and hsCRP) LV parameters (LAD, LVDd and LVEF) over the follow-up period. A first-order autoregressive [AR (1)] working correlation structure was specified to account for within-subject correlations. The model included treatment group (evolocumab vs. statin), time, and their interaction (group × time), adjusting for baseline covariates: age, sex, smoking, drinking, medical history, and postoperative medications. Robust standard errors were applied to ensure valid inference. The group-by-time interaction coefficient (β) was interpreted as the average treatment effect of evolocumab on each biomarker throughout the entire follow-up period. Separate models were fitted for each inflammatory biomarker.

Univariate and multivariate Logistic regression models were established to calculate odds ratios (OR) and 95% confidence intervals (CI) based on whether patients improved global LV function at 12 weeks of treatment. Mediation analysis is used to determine if evolocumab treatment effect on left ventricular global function is mediated through changes in IL-1β and IL-17A levels.

Statistical analysis was performed using SPSS 26.0 and R 4.4.3, and graphs was performed using GraphPad Prism 9.0.0 and R 4.4.3. *P* < 0.05 was considered statistically significant.

## 3 Results

### 3.1 Clinical characters

The mean age of the objects was 57.56 ± 10.56 years, of whom 214 (83.3%) were males, the mean body mass index (BMI) was 25.69 ± 2.94 kg/m^2^.137 (53.3%) of patients had a history of hypertension, 46 (17.9%) of patients had a history of diabetes, 14 of patients (5.4%) had a history of MI. There were 130 (50.6%) active smokers and 82 (31.9%) active drinkers respectively. There was no significant difference between statin group and evolocumab group in general clinical data, past medical history, admission signs, lipid profiles, leukocyte counts, myocardial injury markers, postoperative medication, ultrasound results and angiography results except non-HDL-C and apolipoprotein B (APO B) (Table 1).

**TABLE 1:** Baseline characteristics of the patients with STEMI in different treatments.

Variables	Total (N = 257)	Statin (N = 138)	Evolocumab (N = 119)	*P* value
Age (years)	57.56 ± 10.36	58.92 ± 7.97	56.46 ± 12.17	0.100
Male (n,%)	214 (83.3)	118 (85.5)	96 (80.7)	0.300
BMI(kg/M^**2**^)	25.69 ± 2.94	25.42 ± 3.18	25.98 ± 2.58	0.159
Medical history				
History of hypertension, n (%)	137 (53.3)	78 (56.5)	59 (49.6)	0.266
History of diabetes, n (%)	46 (17.9)	26 (18.8)	20 (16.8)	0.671
Family history of PCAD, n (%)	1 (0.4)	1 (0.7)	0 (0)	0.352
History of MI, n (%)	14 (5.4)	9 (6.5)	5 (4.2)	0.414
History of smoking, n (%)	130 (50.6)	70 (50.7)	60 (50.4)	0.961
History of drinking, n (%)	82 (31.9)	38 (27.5)	44 (37.0)	0.106
Previous PCI, n (%)	22 (8.6)	14 (10.1)	8 (6.7)	0.328
Previous CABG, n (%)	5 (1.9)	2 (1.4)	3 (2.5)	0.535
Statin therapy before admission, n (%)	15 (5.8)	9 (6.5)	6 (5.0)	0.614
Admission				
SBP(mmHg)	120.74 ± 18.97	120.66 ± 19.13	120.83 ± 18.85	0.944
DBP(mmHg)	72.89 ± 13.70	71.19 ± 13.69	74.84 ± 13.49	0.034
HR (bpm)	71.75 ± 11.90	71.44 ± 12.34	72.12 ± 11.42	0.649
Killip class	​	​	​	0.055
I	243 (94.6)	127 (92.0)	116 (97.5)	​
II	14 (5.4)	11 (8.0)	3 (2.5)	​
III	0 (0)	0 (0)	0 (0)	​
IV	0 (0)	0 (0)	0 (0)	​
Killip ≥ II, n, (%)	14 (5.4)	11 (8.0)	3 (2.5)	0.055
Laboratory				
Leukocytes, 10^9^/L	10.17 ± 2.73	10.21 ± 2.62	10.13 ± 2.86	0.827
Neutrophils,%	74.55 ± 8.64	75.37 ± 8.37	73.60 ± 8.88	0.107
Lymphocytes,%	18.13 ± 7.56	17.40 ± 7.44	18.97 ± 7.65	0.103
NLR	4.31 (3.02, 4.69)	4.63 (3.18, 7.05)	3.92 (2.84, 6.27)	0.083
Monocytes,%	6.23 ± 1.93	6.16 ± 1.93	6.31 ± 1.94	0.550
CK_max_ (U/L)	1344.00 (617.00, 2560.00)	1433.00 (679.00, 2429.50)	1172.00 (441.00, 2717.00)	0.304
CK-MB_max_ (U/L)	121.00 (60.25, 227.75)	128.00 (69.00, 218.50)	108.00 (48.00, 236.00)	0.347
hsTnT_max_ (ng/mL)	3.24 (1.38, 7.35)	3.37 (1.78, 7.39)	2.89 (1.15, 7.21)	0.311
NT-proBNP (pg/ml)	140.96 (58.44, 316.43)	142.94 (53.65, 334.92)	138.97 (60.71, 314.35)	0.831
≥140, n (%)	128 (49.8)	71 (51.4)	57 (47.9)	​
<140, n (%)	129 (50.2)	67 (48.6)	62 (52.1)	​
hsCRP (mg/L)	5.00 (1.75, 9.08)	5.09 (1.57, 10.86)	4.95 (2.24, 8.45)	0.742
≥2, n (%)	185 (72.0)	94 (68.1)	91 (76.5)	0.137
≥3, n (%)	163 (63.4)	86 (62.3)	77 (64.7)	0.692
≥5, n (%)	126 (49.0)	69 (50.0)	57 (47.9)	0.737
D-dimer (mg/L)	0.37 (0.26, 0.64)	0.40 (0.26, 0.66)	0.35 (0.25, 0.62)	0.491
Fasting blood glucose (mmol/L)	7.27 ± 2.87	7.10 ± 2.72	7.46 ± 3.03	0.327
eGFR	94.59 (82.66, 105.37)	91.17 (77.73, 104.00)	96.27 (84.84, 106.80)	0.110
Cr (umol/L)	77.99 ± 20.65	80.07 ± 22.29	75.62 ± 18.41	0.087
LDL-C (mmol/L)	3.49 ± 0.62	3.41 ± 0.66	3.58 ± 0.55	0.064
HDL-C (mmol/L)	1.06 ± 0.25	1.07 ± 0.25	1.06 ± 0.24	0.901
VLDL-C (mmol/L)	0.41 (0.23, 0.58)	0.39 (0.23, 0.56)	0.41 (0.23, 0.63)	0.588
Non-HDL-C (mmol/L)	3.91 ± 0.69	3.81 ± 0.71	4.03 ± 0.65	**0.020**
Total cholesterol (mmol/L)	5.03 ± 0.72	4.95 ± 0.74	5.12 ± 0.68	0.091
Triglyceride (mmol/L)	1.62 (1.21, 2.08)	1.53 (1.14, 2.06)	1.71 (1.30, 2.11)	0.242
APO A1 (mmol/L)	1.10 ± 0.20	1.10 ± 0.20	1.10 ± 0.21	0.940
APO B (mmol/L)	1.11 ± 0.17	1.08 ± 0.17	1.16 ± 0.17	**0.002**
Lp(a) (nmol/L)	226.83 (132.52, 363.52)	200.10 (137.17, 319.30)	255.62 (108.318, 404.00)	0.646
LV parameters				
LAD (mm)	37.02 ± 4.26	37.04 ± 4.80	36.99 ± 3.56	0.933
LVDd (mm)	50.96 ± 4.85	51.14 ± 5.45	50.75 ± 4.04	0.521
LVEF (%)	49.47 ± 6.90	49.17 ± 7.04	49.82 ± 6.74	0.453
≥50, n (%)	133 (51.8)	70 (50.7)	63 (52.9)	0.723
<50, n (%)	124 (48.2)	68 (49.3)	56 (47.1)	​
Inflammatory factors				
IL-1β(pg/ml)	16.78 ± 2.57	16.64 ± 2.51	16.95 ± 2.64	0.338
IL-18 (pg/ml)	73.47 ± 10.80	74.00 ± 10.70	72.85 ± 10.92	0.393
IL-17A (pg/ml)	5.68 ± 1.18	5.73 ± 1.11	5.64 ± 1.25	0.544
CCR2(ng/g)	1.65 ± 0.35	1.66 ± 0.37	1.64 ± 0.34	0.691
CAG and treatment				
CAG, n (%)	257 (100)	138 (100)	119 (100)	1
PCI, n (%)	257 (100)	138 (100)	119 (100)	1
Severity of coronary artery lesion, n (%)				
Single-vessel disease, n (%)	72 (28.0)	42 (30.4)	30 (25.2)	0.352
Double-vessel disease, n (%)	85 (33.1)	47 (34.1)	38 (31.9)	0.718
Triple-vessel disease, n (%)	100 (38.9)	49 (35.5)	51 (42.9)	0.228
Left main, n (%)	3 (1.2)	2 (1.4)	1 (0.8)	0.650
Multi-vessel disease, n (%)	185 (72.0)	96 (69.6)	89 (74.8)	0.352
Types of MI	​	​	​	0.773
Anterior, n (%)	107 (41.6)	54 (39.1)	53 (44.5)	​
Inferior, n (%)	85 (33.1)	47 (34.1)	38 (31.9)	​
Anterior and lateral, n (%)	21 (8.2)	13 (9.4)	8 (6.7)	​
Inferior and posterior, n (%)	44 (17.1)	24 (17.4)	20 (16.8)	​
TIMI score	​	​	​	0.123
0, n (%)	3 (1.2)	3 (2.2)	0 (0)	​
1, n (%)	1 (0.4)	1 (0.7)	0 (0)	​
2, n (%)	2 (0.8)	0 (0)	2 (1.7)	​
3, n (%)	251 (97.7)	134 (97.1)	117 (98.3)	​
Postoperative medication, n,(%)				
DAPT	257 (100)	138 (100)	119 (100)	1
Statin	257 (100)	138 (100)	119 (100)	1
ACEI/ARB/ARNI	218 (84.8)	117 (84.8)	101 (84.9)	0.984
β-blocker	201 (78.2)	107 (77.5)	94 (79.0)	0.778

BMI, body mass index; SBP, systolic blood pressure; DBP, diastolic pressure; HR, heart rate; NLR, neutrophil-to-lymphocyte ratio; CK, creatine kinase; CK, creatine kinase-MB: creatine kinase-mb; hs-cTnT, high sensitive-cardiac troponin t; NT-proBNP, n terminal-pro-brain natriuretic peptide; hsCRP, hypersensitive C-reactive protein; eGFR, estimated glomerular filtration rate; Cr, creatinine; LDL-C, low-density lipoprotein cholesterol; HDL-C, high-density lipoprotein cholesterol; VLDL-C, very low-density lipoprotein; APO A1, apolipoprotein A-1; APO B, apolipoprotein B; Lp(a), lipoprotein(a); LAD, left atrial diameter; LVDd, left ventricular end diastolic diameter; LVEF, left ventricular ejection fraction; IL-1β, interleukin-1β; IL-18, interleukin-18; IL-17A, interleukin-17A; CCR2, c-c motif chemokine receptor 2; CAG, coronary angiography; PCI, percutaneous coronary intervention; MI, myocardial infarction; TIMI, thrombolysis in myocardial infarction; DAPT, dual antiplatelet therapy; ACEI, angiotensin converting enzyme inhibitors; ARB, angiotensin receptor blocker; ARNI, angiotensin receptor neprilysin inhibitor. Values are n (%), mean ± SD or median [IQR] for skewed data. Differences were tested using unpaired Mann Whitney test, unpaired t-test or the Chi-square test as appropriate.

The bold values indicate there were differences between the two groups.

### 3.2 Effect of evolocumab on lipid lowering in patients with STEMI

**FIGURE 2:**
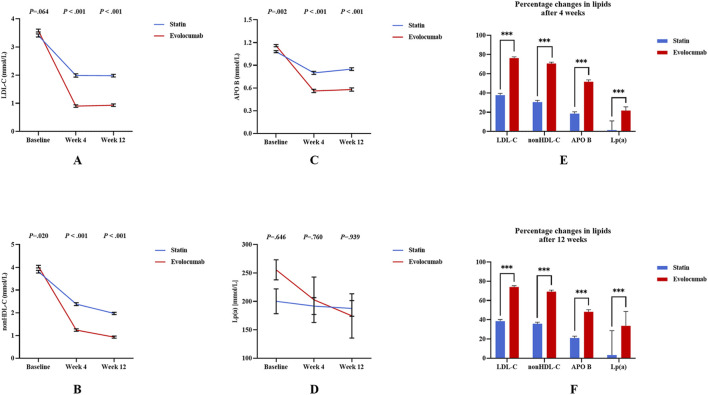
Differences in lipids for PCSK9i and Statins in 257 STEMI patients. **(A–D)** Show the changes of lipids at baseline, week four and week 12 of the two groups; **(E,F)** Show the comparison of percentage changes of lipids. Error bars indicate standard error of mean. Differences were tested using the unpaired t-test. ^*^ *P* < 0.05 ^**^ *P* < 0.01 ^***^ *P* < 0.001.

LDL-C levels in the evolocumab group decreased significantly from 3.58 mmol/L to 0.90 mmol/L after 4 weeks of treatment with a decrease of 76.26% (*P* < 0.001) and remained stable at week 12 (Supplementary Table S1; Figure 2A). Non-HDL-C levels in the evolocumab group decreased significantly from 4.03 mmol/L to 1.24 mmol/L after 4 weeks of treatment with a decrease of 70.59% (*P* < 0.001) and remained stable at week 12 (Supplementary Table S1; Figure 2B). APO B levels in the evolocumab group decreased significantly from 1.16 mmol/L to 0.56 mmol/L after 4 weeks of treatment with a decrease of 51.63% (*P* < 0.001) and remained stable at week 12 (Supplementary Table S1; Figure 2C). Lipoprotein (a) [Lp(a)] levels in the evolocumab group decreased significantly from 255.62 nmol/L to 202.77 mmol/L after 4 weeks of treatment with a decrease of 21.83% (*P* < 0.001), continued to decreased significantly to 174.44 mmol/L at week 12 with a decrease of 33.67% (*P* < 0.001) (Supplementary Table S1; Figure 2D). Furthermore, the levels of LDL-C, non-HDL-C, and APO B in the evolocumab group were significantly lower than those in the statin group at 4 and 12 weeks of treatment (Figures 2A–D). The percentage changes of LDL-C, non-HDL-C, APO B, and Lp(a) in the evolocumab group were significantly higher than those in the statin group at 4 and 12 weeks of treatment (Supplementary Table S1; Figures 2E,F).

### 3.3 Effect of evolocumab on inflammatory factors in patients with STEMI

**FIGURE 3:**
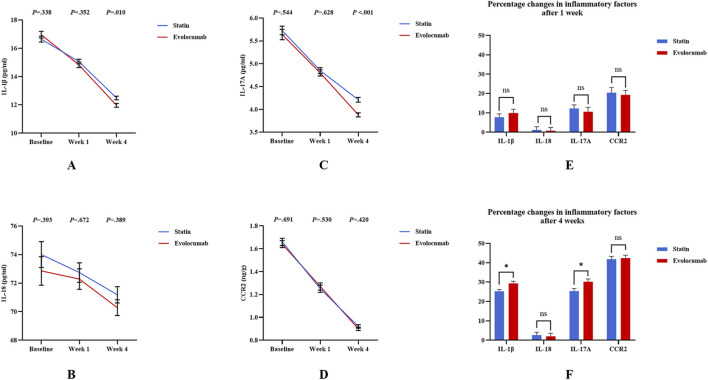
Differences in inflammatory factors for PCSK9i and Statins in 257 STEMI patients. **(A–D)** Show the changes of inflammatory factors at baseline, week 1 and week 4 of the two groups; **(E,F)** Show the comparison of percentage changes of inflammatory factors. Error bars indicate standard error of mean. Differences were tested using the unpaired t-test. ^*^ *P* < 0.05 ^**^ *P* < 0.01 ^***^ *P* < 0.001.

Compared to baseline, there was a reduction in IL-1β in both evolocumab and statin groups at 1 week and maintained at 4 weeks (Supplementary Table S2). Percentage change in IL-1β from baseline to 1 week was −9.89% ± 21.67% in the evolocumab group (from mean 16.95 to 14.83 mmol/L) versus −7.79% ± 19.35% in the statin group (from mean 16.64 to 15.06 mmol/L), with no difference between groups (*P* = 0.412) (Figures 3A,E). Percentage change in IL-1β from baseline to 4 weeks was −29.35% ± 12.08% in the evolocumab group (from mean 16.95 to 11.96 mmol/L) versus −25.27% ± 10.89% in the statin group (from mean 16.64 to 12.48 mmol/L), amounting to a mean difference of −4.08% between groups (95% confidence interval [CI]: −7.24% to −0.91%; *P* = 0.012) (Figures 3A,F).

Compared to baseline, there was a reduction in IL-17A in both evolocumab and statin groups at 1 week and maintained at 4 weeks (Supplementary Table S2). Percentage change in calculated IL-17A from baseline to 1 week was −10.60% ± 24.31% in the evolocumab group (from mean 5.64 to 4.80 mmol/L) versus −12.28% ± 22.11% in the statin group (from mean 5.73 to 4.85 mmol/L), with no difference between groups (*P* = 0.562) (Figures 3C,E). Percentage change in IL-17A from baseline to 4 weeks was −30.22% ± 15.91% in the evolocumab group (from mean 5.64 to 3.88 mmol/L) versus −25.35% ± 15.91% in the statin group (from mean 5.73 to 4.21 mmol/L), amounting to a mean difference of −4.88% between groups (95% confidence interval [CI]: −9.06% to −0.69%; *P* = 0.023) (Figures 3C,F).

Compared to baseline, both the evolocumab and statin groups showed significant reductions in peripheral blood CCR2 levels at 1 week and 4 weeks, with no statistically significant differences observed between groups (Supplementary Table S2; Figure 3D). For IL-18 and hsCRP levels, neither group demonstrated significant changes from baseline to 1 week after treatment, and no intergroup differences were observed (Supplementary Table S2). However, by 4 weeks post-treatment, both groups exhibited significant reductions in IL-18 and hsCRP levels compared to baseline, though again without significant differences between the treatment groups (Supplementary Table S2; Figure 3B).

To explore the effect of evolocumab treatment on inflammatory-immune factors in patients with different LVEF, we further compared patients into groups according to whether their LVEF≥ 50%. The results showed that after 4 weeks of treatment in patients with LVEF<50%, evolocumab treatment significantly reduced the levels of IL-1β and IL-17A compared with the statin group (−29.87% ± 11.40% vs −24.37% ± 10.49%, *P* = 0.011; −31.67% ± 14.96% vs −23.01% ± 16.84%, *P* = 0.041), while this difference was not found in patients with LVEF ≥ 50% (Supplementary Table S3).

### 3.4 Effect of evolocumab on early left ventricular function in patients with STEMI

**FIGURE 4:**
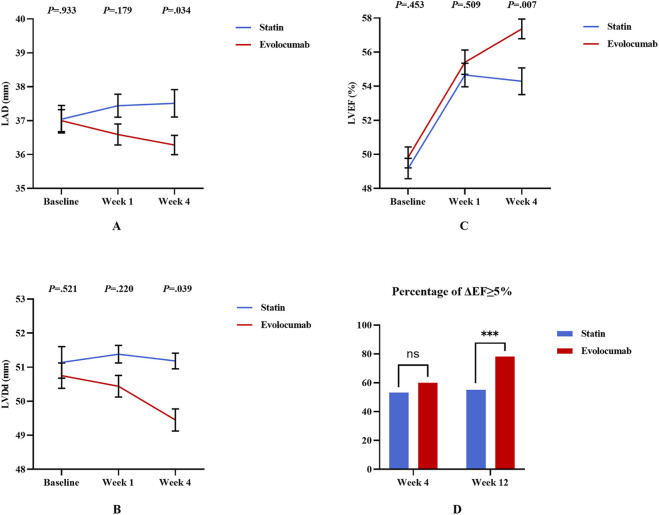
Differences in LV function for PCSK9i and Statins in 257 STEMI patients. **(A–C)** Show the changes of LV function parameters at baseline, week 4 and week 12 of the two groups; **(D)** Shows the comparison of proportion of patients with ΔEF≥ 5% at week 12. Error bars indicate standard error of mean. Differences were tested using the unpaired t-test and chi-square test. ^*^ *P* < 0.05 ^**^ *P* < 0.01 ^***^ *P* < 0.001.

Compared to baseline, there was no difference at 4 weeks in LAD in both evolocumab and statin groups (Supplementary Table S4; Figure 4A). At 12 weeks, there was a reduction in LAD in evolocumab group (Supplementary Table S4). Absolute change in LAD from baseline to 12 weeks was −0.94 ± 3.51 mm in the evolocumab group (from mean 37.00 mm–36.28 mm) versus 0.29 ± 3.70 mm in the statin group (from mean 37.04 mm–37.51 mm), amounting to a mean difference of −1.22 mm between groups (95% confidence interval [CI]: −2.35 mm to −0.10 mm; *P* = 0.034) (Supplementary Table S5; Figure 4A).

Compared to baseline, there was no difference at 4 weeks in LVDd in both evolocumab and statin groups (Supplementary Table S4; Figure 4B). At 12 weeks, there was a reduction in LVDd in both evolocumab and statin groups (Supplementary Table S4). Absolute change in LVDd from baseline to 12 weeks was −0.94 ± 3.51 mm in the evolocumab group (from mean 50.75 mm–49.45 mm) versus 0.29 ± 3.70 mm in the statin group (from mean 51.14 mm–51.18 mm), amounting to a mean difference of −1.31 mm between groups (95% confidence interval [CI]: −2.55 mm to −0.63 mm; *P* = 0.028) (Supplementary Table S5; Figure 4B).

Compared to baseline, LVEF increased significantly in both evolocumab and statin groups at 4 weeks and maintained at 12 weeks (Supplementary Table S4; Figure 4C). Absolute change in LVEF from baseline to 4 weeks was 5.38% ± 7.62% in the evolocumab group (from mean 49.82%–55.41%) versus 5.19% ± 6.53% in the statin group (from mean 49.82%–55.41%), with no difference between groups (*P* = 0.852) (Supplementary Table S4; Figure 4C). Absolute change in LVEF from baseline to 12 weeks was 7.53% ± 7.44% in the evolocumab group (from mean 49.82%–57.36%) versus 5.07% ± 8.05% in the statin group (from mean 49.17%–54.29%),amounting to a mean difference of −2.52% between groups (95% confidence interval [CI]: −9.59% to −2.02%; *P* = 0.030) (Supplementary Table S5; Figure 4C).

To explore the effect of evolocumab treatment on LV function in patients with different LVEF, we further compared patients into groups according to whether their LVEF%≥50%. The results showed that after 12 weeks of treatment in patients with LVEF < 50%, absolute change in LAD (0.40 ± 2.96 vs −0.71 ± 3.17, *P* = 0.049), LVDd (−0.23 ± 3.02 vs −1.62 ± 3.21, *P* = 0.045) and LVEF (7.40 ± 8.07 vs 11.36 ± 7.19, *P* = 0.014) in evolocumab group different from that in statin group, while this difference was not found in patients with LVEF ≥ 50% (Supplementary Table S5).

At 4 weeks, the percentage of ΔEF≥ 5% was 60.0% in evolocumab group versus 53.3% in statin group, with no significant difference between groups (*P* = 0.858). At 12 weeks, the percentage of ΔEF ≥ 5% was 78.2% in evolocumab group versus 55.1% in statin group, with significant difference between groups (*P* < 0.001) (Figure 4D).

After 12 weeks of treatment, patients with ΔEF ≥ 5% had higher proportion of NT-proBNP ≥ median (140 pg/ml) (54.4% vs 45.6%, *P* = 0.040), lower D-dimer levels [0.35 (0.25,0.60) vs 0.44 (0.31,0.80), *P* = 0.024], higher LVDd (51.52 ± 4.99 vs 49.88 ± 4.39, *P* = 0.010), lower IL-1β levels (16.51 ± 2.65 vs 17.30 ± 2.35, *P* = 0.018), lower LVEF (48.44 ± 6.59 vs 51.45 ± 7.07, *P* = 0.001) and higher proportion of LVEF<50% (56.6% vs 34.1%, *P* = 0.001) on admission. In addition, patients with ΔEF ≥ 5% had a higher level of changes in IL-1β at week 4 (−5.20 ± 2.65 vs −4.13 ± 2.04, *P* = 0.003), IL-17A at week 4 (1.90 ± 1.14 vs 1.46 ± 1.20, *P* = 0.008) and LDL-C at week 4 (−2.19 ± 1.24 vs −1.66 ± 1.20, *P* < 0.001) (Table 2).

**TABLE 2:** Baseline characteristics of the patients stratified by ΔEF at week 12.

Variables	Total (N = 257)	ΔEF<0.05 (N = 88)	ΔEF≥0.05 (N = 169)	*P* value
Age (years)	57.56 ± 10.36	58.97 ± 10.01	56.87 ± 10.49	0.051
Male, n (%)	214 (83.3)	73 (83.0)	141 (83.4)	0.922
BMI(kg/M^**2**^)	25.69 ± 2.94	25.53 ± 3.21	25.77 ± 2.79	0.364
Medical history				
History of hypertension, n (%)	137 (53.3)	47 (53.4)	90 (53.3)	0.981
History of diabetes, n (%)	46 (17.9)	16 (18.2)	30 (17.8)	0.932
Family history of CVD, n (%)	1 (0.4)	1 (1.1)	0 (0)	0.165
History of MI, n (%)	14 (5.4)	6 (6.8)	8 (4.7)	0.485
History of smoking, n (%)	130 (50.6)	43 (48.9)	87 (51.5)	0.691
History of drinking, n (%)	82 (31.9)	28 (31.8)	54 (32.0)	0.982
Previous PCI, n (%)	22 (8.6)	8 (9.1)	14 (8.3)	0.826
Previous CABG, n (%)	5 (1.9)	0 (0)	5 (3.0)	0.103
Statin therapy before admission, n (%)	15 (5.8)	6 (6.8)	9 (5.3)	0.628
Admission				
SBP(mmHg)	120.74 ± 18.97	123.26 ± 20.82	119.40 ± 17.83	0.123
DBP(mmHg)	72.89 ± 13.70	72.91 ± 13.51	72.87 ± 13.83	0.984
HR (bpm)	71.75 ± 11.90	70.89 ± 12.40	72.21 ± 13.83	0.400
Killip class	​	​	​	0.905
I	243 (94.6)	83 (94.3)	160 (94.7)	​
II	14 (5.4)	5 (5.7)	9 (5.3)	​
III	0 (0)	0 (0)	2 (1.0)	​
IV	0 (0)	0 (0)	0 (0)	​
Laboratory				
Leukocytes, 10^9^/L	10.17 ± 2.73	10.44 ± 2.98	10.03 ± 2.58	0.263
Neutrophils,%	74.55 ± 8.64	75.62 ± 7.58	73.99 ± 9.12	0.157
Lymphocytes,%	18.13 ± 7.56	17.40 ± 6.92	18.51 ± 7.87	0.270
NLR	4.31 (3.02,4.69)	4.57 (3.28,6.76)	4.23 (2.80,6.69)	0.333
Monocytes,%	6.23 ± 1.93	6.10 ± 1.89	6.30 ± 1.96	0.434
CK_max_ (U/L)	1344.00 (617.00, 2560.00)	1281.00 (684.00, 2603.00)	1492.00 (516.00, 2511.00)	0.456
CK-MB_max_ (U/L)	121.00 (60.25, 227.75)	109.00 (65.00,267.00)	125.00 (53.00, 214.50)	0.613
hsTnT_max_ (ng/mL)	3.24 (1.38,7.35)	3.43 (1.49,7.14)	3.06 (1.11, 7.39)	0.789
NT-proBNP (pg/ml)	140.96 (58.44, 316.43)	110.08 (48.09, 244.67)	144.45 (54.22, 369.50)	0.098
≥140, n (%)	128 (49.8)	36 (40.9)	92 (54.4)	**0.040**
<140, n (%)	129 (50.2)	52 (59.1)	77 (45.6)	-
hsCRP (mg/L)	5.00 (1.75,9.08)	5.04 (1.54,8.06)	5.00 (1.85,9.39)	0.477
≥2, n (%)	185 (72.0)	61 (69.3)	124 (73.4)	0.492
≥3, n (%)	163 (63.4)	51 (58.0)	112 (66.3)	0.189
≥5, n (%)	126 (49.0)	43 (48.9)	83 (49.1)	0.970
D-dimer (mg/L)	0.37 (0.26,0.64)	0.44 (0.31,0.80)	0.35 (0.25,0.60)	**0.024**
Fasting blood glucose (mmol/L)	7.27 ± 2.87	6.59 (5.35,7.68)	6.34 (5.57,8.22)	0.778
eGFR	94.59 (82.66, 105.37)	93.43 (82.67, 103.54)	95.80 (82.63, 105.84)	0.218
Cr (umol/L)	77.99 ± 20.65	78.79 ± 18.94	77.59 ± 21.52	0.660
LDL-C (mmol/L)	3.49 ± 0.62	3.49 ± 0.67	3.49 ± 0.59	0.998
Δ LDL-C at week 4 (mmol/L)	−2.02 ± 1.24	−1.66 ± 1.20	−2.19 ± 1.24	**<0.001**
LDL-C<1.4 mmol/L at week 4 (n,%)	116 (45.1)	42 (47.7)	74 (43.8)	0.547
HDL-C (mmol/L)	1.06 ± 0.25	1.08 ± 0.26	1.06 ± 0.24	0.583
VLDL-C (mmol/L)	0.41 (0.23, 0.58)	0.39 (0.22, 0.60)	0.41 (0.24, 0.58)	0.744
Non-HDL-c (mmol/L)	3.91 ± 0.69	3.89 ± 0.74	3.92 ± 0.67	0.802
Total cholesterol (mmol/L)	5.03 ± 0.72	5.05 ± 0.79	5.03 ± 0.68	0.823
Triglyceride (mmol/L)	1.62 (1.21, 2.08)	1.53 (1.15, 2.01)	1.64 (1.21, 2.12)	0.305
APO A1 (mmol/L)	1.10 ± 0.20	1.08 ± 0.22	1.10 ± 0.19	0.408
APO B (mmol/L)	1.11 ± 0.17	1.11 ± 0.17	1.12 ± 0.18	0.653
Lp(a)	226.83 (132.52, 363.52)	218.05 (137.00, 352.96)	228.19 (101.35, 392.00)	0.995
UCG parameters				
LAD (mm)	37.02 ± 4.26	36.80 ± 3.72	37.13 ± 4.52	0.551
LVDd (mm)	50.96 ± 4.85	49.88 ± 4.39	51.52 ± 4.99	**0.010**
LVEF (%)	49.47 ± 6.90	51.45 ± 7.07	48.44 ± 6.59	**0.001**
≥50, n (%)	133 (51.8)	58 (65.9)	75 (44.4)	**0.001**
<50, n (%)	124 (48.2)	30 (34.1)	94 (56.6)	**-**
Inflammatory factors				
IL-1β(pg/ml)	16.78 ± 2.57	17.30 ± 2.35	16.51 ± 2.65	**0.018**
Δ IL-1β at week 4 (pg/ml)	−4.82 ± 2.50	−4.13 ± 2.04	−5.20 ± 2.65	**0.003**
IL-18 (pg/ml)	73.47 ± 10.80	73.69 ± 10.47	73.36 ± 10.99	0.816
Δ IL-18 at week 4 (pg/ml)	−2.64 ± 12.46	−2.78 ± 11.79	−2.56 ± 12.88	0.915
IL-17A (pg/ml)	5.68 ± 1.18	5.76 ± 1.16	5.64 ± 1.19	0.443
Δ IL-17A at week 4 (pg/ml)	−1.73 ± 1.18	−1.46 ± 1.20	−1.90 ± 1.14	**0.008**
CCR2(ng/g)	1.65 ± 0.35	1.63 ± 0.39	1.66 ± 0.33	0.551
Δ CCR2 at week 4 (ng/g)	−0.74 ± 0.39	−0.73 ± 0.42	−0.74 ± 0.38	0.754
CAG and treatment				
CAG, n (%)	257 (100)	103 (100)	199 (100)	1
PCI, n (%)	257 (100)	103 (100)	199 (100)	1
Severity of coronary artery lesion, n (%)				
Single-vessel disease, n (%)	72 (28.0)	26 (29.5)	46 (27.2)	0.694
Double-vessel disease, n (%)	85 (33.1)	33 (37.5)	52 (30.8)	0.276
Triple-vessel disease, n (%)	100 (38.9)	29 (33.0)	71 (42.0)	0.158
Left main, n (%)	3 (1.2)	0 (0.0)	3 (1.8)	0.209
Multi-vessel disease, n (%)	185 (72.0)	62 (70.5)	123 (72.8)	0.694
Types of MI	​	​	​	0.242
Anterior, n (%)	107 (41.6)	29 (33.0)	78 (46.2)	​
Inferior, n (%)	85 (33.1)	34 (38.6)	51 (30.2)	​
Anterior and lateral, n (%)	21 (8.2)	8 (9.1)	13 (7.6)	​
Inferior and posterior, n (%)	44 (17.1)	17 (19.3)	27 (16.0)	​
TIMI score	​	​	​	0.307
0, n (%)	3 (1.2)	2 (2.3)	1 (0.6)	​
1, n (%)	1 (0.4)	1 (1.1)	0 (0)	​
2, n (%)	2 (0.8)	1 (1.1)	1 (0.6)	​
3, n (%)	251 (97.6)	84 (95.5)	167 (98.8)	​
Postoperative medication, n (%)				
DAPT	257 (100)	103 (100)	199 (100)	1
Statin	257 (100)	103 (100)	199 (100)	1
ACEI/ARB/ARNI	218 (84.8)	73 (83.0)	145 (85.8)	0.546
β-blocker	201 (78.2)	63 (71.6)	138 (81.7)	0.064
PCSK9i	119 (46.3)	26 (29.5)	93 (55.0)	**<0.001**

BMI, body mass index; SBP, systolic blood pressure; DBP, diastolic pressure; HR, heart rate; NLR, neutrophil-to-lymphocyte ratio; CK, creatine kinase; CK, creatine kinase-MB: creatine kinase-mb; hs-cTnT, high sensitive-cardiac troponin t; NT-proBNP, n terminal-pro-brain natriuretic peptide; hsCRP, hypersensitive C-reactive protein; eGFR, estimated glomerular filtration rate; Cr, creatinine; LDL-C, low-density lipoprotein cholesterol; HDL-C, high-density lipoprotein cholesterol; VLDL-C, very low-density lipoprotein; APO A1, apolipoprotein A-1; APO B, apolipoprotein B; Lp(a), lipoprotein (a); LAD, left atrial diameter; LVDd, left ventricular end diastolic diameter; LVEF, left ventricular ejection fraction; IL-1β, interleukin-1β; IL-18, interleukin-18; IL-17A, interleukin-17A; CCR2, c-c motif chemokine receptor 2; CAG, coronary angiography; PCI, percutaneous coronary intervention; MI, myocardial infarction; DAPT, dual antiplatelet therapy; TIMI, thrombolysis in myocardial infarction; ACEI, angiotensin converting enzyme inhibitors; ARB, angiotensin receptor blocker; ARNI, angiotensin receptor neprilysin inhibit. Values are n (%), mean ± SD or median [IQR] for skewed data. Differences were tested using unpaired Mann Whitney test, unpaired t-test or the Chi-square test as appropriate.

The bold values indicate there were differences between the two groups.

### 3.5 Generalized estimating equation analysis of evolocumab effects

To evaluate the longitudinal association between evolocumab treatment and changes in inflammatory biomarker levels and LV function parameters over the study period, we employed a Generalized Estimating Equations (GEE) model. This approach was selected to account for the inherent within-subject correlation arising from repeated measurements across multiple time points.

The GEE analysis revealed a significant association between evolocumab treatment and reductions in IL-1β and IL-17A levels. Specifically, the active treatment group exhibited a statistically significant decrease in IL-1β (*β* = −0.18, 95% CI = −0.31 to −0.04, *P* = 0.012) and IL-17A (*β* = −0.13, 95% CI = −0.01 to −0.34, *P* = 0.030) compared to the statin group over the 4-week follow-up period (Supplementary Table S6). There was no significant correlation between evolocumab treatment and IL-18, CCR2, and hsCRP levels in patients with STEMI after treatment (Supplementary Table S6).

The GEE analysis revealed significant associations between evolocumab treatment and improvements in all three LV function parameters compared to the statin group over the 12-week follow-up period. Active treatment was associated with a significant increase in LVEF (*β* = 0.18, 95% CI = 0.01 to 0.34, *P* = 0.036) and a significant reduction in LVDd (*β* = −0.10, 95% CI = −0.19 to 0.00, *P* = 0.042) (Supplementary Table S6). Furthermore, a significant decrease in LAD was also observed (*β* = −0.12, 95% CI = −0.22 to −0.02, *P* = 0.019) (Supplementary Table S7).

In order to exclude the impact of other medications on inflammatory factors, we conducted multivariant and univariant analysis through GEE to evaluate the relationship between the use of -blocker and ACEI/ARB/ARNI and inflammatory factors. Multivariate analysis results showed that none of the above drugs was found to be related to the concentration of IL-1β, IL-18, IL-17A, and CCR2 (Supplementary Table S8).

### 3.6 Logistic regression analysis of global left ventricular function improvement

**FIGURE 5:**
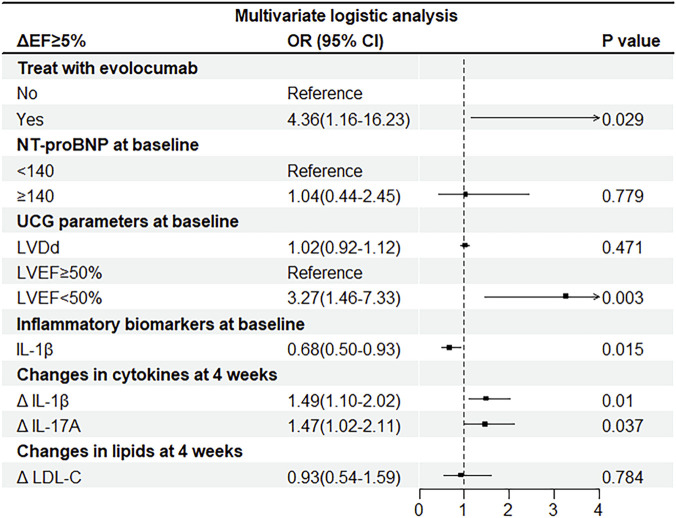
Multivariate logistic regression analysis of ΔEF≥5% in from the two study groups in 257 STEMI patients. LVDd, left ventricular end diastolic diameter; LVEF, left ventricular ejection fraction; IL-17A, interleukin-17A; IL-1β, interleukin-1β; LDL-C, low-density lipoprotein cholesterol; PCSK9i, proprotein convertase subtilisin/kexin type 9 (PCSK9) inhibitor; OR, odds ratio. Changes (Δ) were obtained by calculating the differences between the levels of the inflammatory factors or LDL-C at baseline and after 4 weeks.

Univariate logistic regression analysis was performed with ΔEF ≥ 5% at 12 weeks in STEMI patients as the dependent variable, and evolocumab treatment, reduction of inflammatory factors at 4 weeks, reduction of LDL-C at 4 weeks and 12 weeks, and baseline covariates as independent variables. Variables with *P* < 0.05 in univariate logistic regression analysis except reduction in LDL-C at 4 weeks were included in multivariate logistic regression analysis. The multivariate logistic regression analysis showed that evolocumab treatment (OR = 4.36, 95% CI = 1.16–16.23, *P* = 0.029), IL-1β reduction (OR = 1.49, 95% CI = 1.10–2.02, *P* = 0.010) and IL-17A reduction (OR = 1.47, 95% CI = 1.02–2.11, *P* = 0.037) after 4 weeks of treatment, LVEF < 50% (OR = 3.27, 95% CI = 1.46–7.33, *P* = 0.003), and baseline IL-1β level (OR = 0.68, 95% CI = 0.50–0.93, *P* = 0.015) were independent risk predictors of ΔEF ≥ 5% in STEMI patients (Figure 5).

### 3.7 Mediating effect analysis of evolocumab on improving global left ventricular function in patients with STEMI

In order to explore whether the effect of evolocumab on early cardiac function in patients with STEMI is mediated through its effect on inflammatory factors, we conducted a mediation analysis using evolocumab treatment as the independent variable, ΔEF ≥ 5% at 12 weeks as the dependent variable, and changes in IL-1β (ΔIL-1β) and IL-17A (ΔIL-17A) levels at 4 weeks as mediating variables.

**FIGURE 6:**
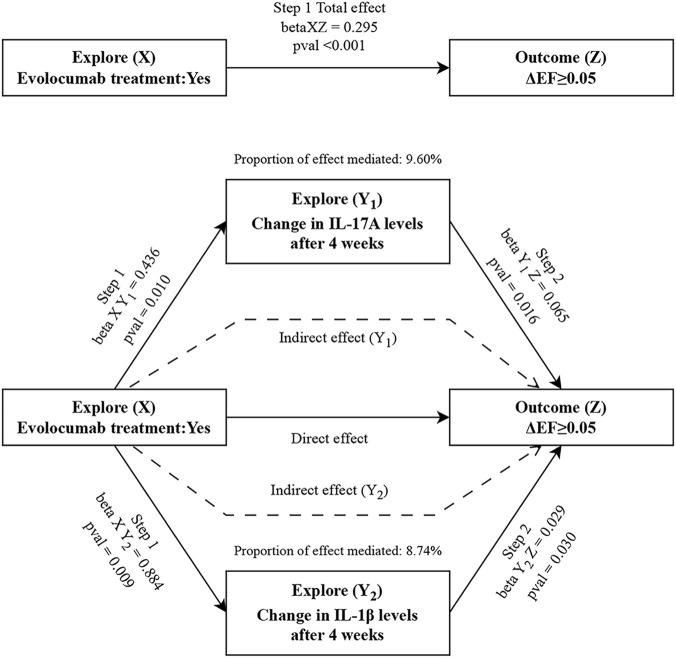
Mediation analysis of the effect of evolocumab treatment on ΔEF≥5% via inflammatory factors in 257 STEMI patients from the two study groups.

The results showed that Change in IL-1β level at 4 weeks, as a potential mediator, increased intensity of evolocumab treatment in promoting ΔEF ≥ 5% at 12 weeks (mediating effect: 8.74%). Similarly, change in IL-17A level at 4 weeks increased intensity of evolocumab treatment in promoting ΔEF ≥ 5% at 12 weeks (mediating effect: 9.60%) (Figure 6).

## 4 Discussion

In the current study, we sought to assess the effects of early evolocumab treatment on acute inflammation and early left ventricular function, we found: 1) IL-1β and IL-17A were significantly reduced after 1 week and 4 weeks of evolocumab treatment compared with baseline; 2) After 4 weeks of evolocumab treatment, IL-1β and IL-17A levels and their reduction were significantly different from those of statin alone; 3) LVEF in evolocumab group was significantly higher than that in statin group at 12 weeks of treatment, and the proportion of patients with ΔEF ≥ 5% was significantly higher than that in statin group; 4) The effects of evolocumab on early left ventricular function are mediated in part by its effects on IL-1β and IL-17A.

Previous large-scale studies, such as the FOURIER trial and ODYSSEY trial, focused on the effects of PCSK9i on LDL-C levels and long-term cardiovascular outcomes. On this basis, the EVOPACS trial assessed the feasibility, safety, and LDL-C lowering efficacy of evolocumab initiated during the in-hospital phase of ACS. In addition, EVOPACS trial investigated changes in hsCRP, IL-1β, and IL-6 at 8 weeks in ACS patients. However, the results showed no significant differences in these inflammatory biomarkers compared with statin treatment alone (Koskinas et al., 2019). To further explore the effect of early evolocumab treatment on inflammation and immune in STEMI patients, we focused on changes in earlier inflammation-immune factors (changes in hsCRP, IL-1β, IL-18, IL-17A, and CCR2 after 1 week and 4 weeks).

Consistent with previous studies, we observed no significant differences in hsCRP levels and magnitude of decrease compared with statin treatment alone, either after 1 and 4 weeks of evolocumab treatment. However, our results suggest that evolocumab may affect inflammation and immunity in patients by acting on the IL-1β/IL-17A axis rather than hsCRP. Inflammation and immunity play a crucial role in ventricular remodeling following STEMI. The post- STEMI healing and cardiac remodeling processes are characterized by intense intramyocardial inflammatory responses (Miñana et al., 2020). In this process, the interleukin family plays a key role (Abbate et al., 2011). In the induced model of STEMI, mRNA levels of pro-inflammatory factors such as IL-1β increased significantly at 1 week after the onset of disease and gradually decreased over the following 3 weeks, but the levels of inflammatory cytokines in the heart remain elevated at 4 weeks. Studies have shown that, regardless of LVEF, patients with low IL-1β levels have a significantly reduced risk of cardiac death and heart failure-related outcomes compared with patients with high IL-1β levels. IL-1β can work in concert with IL-23 to drive IL-17A production in γδT cells. IL-17A can activate inflammation, promote the release of pro-inflammatory factors such as cytokines IL-6, and promote the release of chemokines CXCL1, CCL2, etc (Luo et al., 2022). Existing studies have shown that IL-17A expression is elevated in the left ventricular infarction area. IL-17A reaches its peak at 7 days after myocardial infarction and remains at a high level until 14 days thereafter, suggesting that this inflammatory pathway may play a role in the early stages of cardiac remodeling after myocardial infarction (Mora-Ruíz et al., 2019). During ventricular remodeling, IL-17A also facilitates recruitment of CCR2+ monocytes to infarct zones, thereby exacerbating maladaptive remodeling (Ávalos et al., 2012). In our multivariate logistic regression model, patients with higher IL-1β at baseline (OR = 0.72, 95% CI = 0.55–0.93, *P* = 0.013) were less likely to have a ΔEF ≥ 5% after 12 weeks, further indicating an effect of inflammation on early left ventricular function in STEMI patients.

Given the role of IL-1β/IL-17A in the early stages of cardiac remodeling, we considered that evolocumab may have a beneficial effect on early left ventricular function in patients with STEMI. Therefore, we explored differences in LVEF, LVDd, and LAD between evolocumab and statin groups at 4 and 12 weeks of treatment. The results showed that the reductions in LVDd and LAD and the increase in LVEF in evolocumab group were significantly greater than those in statin group at 12 weeks. The proportion of patients with ΔEF ≥ 5% was significantly higher than that in statin group. The above results suggest that evolocumab may alleviate the changes of early left heart structure in patients with STEMI, and then increase LVEF and improve left ventricular function. To further examine whether evolocumab’s improvement in early LV function was associated with its inhibition of IL-1β/IL-17A, we performed a mediating effect analysis, which showed that the effect of evolocumab on early LV function was probably mediated in part by its inhibition of IL-1β/IL-17A axis.

PCI is one of the established treatments for patients with STEMI. Although the incidence of heart failure in STEMI patients has declined over the past few years due to reperfusion therapy, the incidence of heart failure after PCI in STEMI patients remains between 14% and 36% in different studies, and patient mortality seems to be associated with left ventricular systolic dysfunction (Gharagozloo et al., 2024; Hellermann et al., 2002). A 5% decrease in LVEF measured by ventriculography during hospitalization for MI increases the risk of heart failure after discharge by 12%–18%. Similarly, a 5% decrease in LVEF assessed by echocardiography at 5–20 months after MI increases the risk of heart failure by 20% (Chong et al., 2018; Kelly et al., 2011; Shih et al., 2019). Patients with both heart failure and left ventricular systolic dysfunction face significantly elevated risks of adverse clinical outcomes, including cardiac rupture, cardiac arrest, stroke, prolonged hospitalization, ventricular arrhythmias, recurrent myocardial infarction, and mortality (Lewis et al., 2008). The prevention of post-PCI heart failure in STEMI patients is a widely concerned issue in clinical practice (Jingxuan et al., 2023).

Our results suggest that PCSK9i therapy can early reduce inflammatory factors and improve early left ventricular function especially in the patients with LVEF < 50%. These effects may change the strategy and timing of PCSK9i treatment, rather than as a simple lipid-lowering therapy. In addition, our study also provides another therapeutic idea, that is, to target inflammatory factors such as IL-1β and IL-17A, which needs further exploration in more trials.

Most of the clinical research results on the effects of PCSK9i on inflammatory factors and LV function in STEMI patients are based on the conclusions drawn in small sample studies, and there is a lack of large-scale clinical trial data as a basis. On this basis, we conducted this single-center, and non-interventional Real-World study. In addition, unlike existing studies focusing on the lipid-lowering effects and long-term cardiovascular outcomes of PCSK9i treatment, our study focuses on the early application of PCSK9i and its early effects on inflammation-immune and left ventricular function in STEMI patients. Our study reveals for the first time that early use of PCSK9i can rapidly inhibit IL-1β/IL-17A and improve LV function within 12 weeks. Another highlight of our study is that we linked the effects of PCSK9i on inflammation-immune and LV function through mediating effect analysis, proposing a new mechanism by which PCSK9i mediates cardiac function improvement through inflammation-immune. To our knowledge, this is the first trial to demonstrate that the cardioprotective effect of PCSK9i is partially mediated by IL-1β/IL-17A inhibition, while independent of the reduction of LDL-C. This challenges the traditional concept that the cardiac protection effect of PCSK9i is mainly achieved through lipid reduction. In the mediation effect analysis results, changes in IL-1β and IL-17A mediated 8.74% and 9.60% of the effects respectively, which means that the effect of PCSK9i on early cardiac function in STEMI patients may also be mediated through other factors. PCSK9 influences fatty acid uptake, inflammatory pathways, and mitochondrial function through its interactions with key signaling mediators such as CD36 and Toll-like receptor 4 (TLR4), exerting profound effects on myocardial homeostasis (Wang et al., 2025). A study shows that PCSK9i directly improves cardiomyocyte and endothelial cell bioenergetics by improving mitochondrial respiration and membrane potential, thereby improving myocardial cell function (Braczko et al., 2023). In addition, PCSK9i bear intrinsic anti-inflammatory, anti-autophagic, and antioxidant properties in endothelial cells, which might be mediated by the NAD-dependent deacetylase sirtuin-3 (SIRT3) (D'Onofrio et al., 2023). The loss of SIRT3 has been confirmed to be associated with myocardial inflammation, myocardial fibrosis and myocardial hypertrophy (Palomer et al., 2020; Peng et al., 2024). PCSK9 inhibits the expression of SIRT3, thereby enhancing the inflammatory response, oxidative stress and mitochondrial autophagy during myocardial cell injury, causing myocardial remodeling, which suggests that PCSK9i might improve cardiac function through this pathway (Kaur et al., 2026). Early application of PCSK9i can quickly reduce tumor necrosis factor-α (TNF-α), IL-6 and other inflammatory factors in patients with ACS (Ou et al., 2022), and these inflammatory factors may also have potential effects on patients ‘cardiac remodeling (Hilgendorf et al., 2024). All of these potential possibilities need to be further confirmed.

In order to ensure the reliability of the test results, we took into account the impact of time on inflammatory factors and left ventricular function, so we introduced generalized regression equations to further correct and test the results of the study.

While providing important insights, this study has several limitations: First, the trial is a single-center study and moderate sample size (N = 257) may affect generalizability. In addition, inflammatory marker analysis was limited to selected cytokines (IL-1β, IL-18, IL-17A, CCR2), potentially missing other relevant pathways. Furthermore, although echocardiography was performed using the proper methodology, it is subject to operator-dependent variability. Cardiovascular magnetic resonance would have provided more precise assessment. These limitations highlight the need for larger, multicenter trials with longer follow-up, broader biomarker panels, and clinical endpoint evaluation to confirm these promising findings and establish optimal treatment protocols. In the future, the inflammation-immune mechanism underlying the action of PCSK9 inhibitors needs to be further explored.

## 5 Conclusion

In summary, this study provides robust evidence for the current results of the early application of PCSK9 inhibitors on inflammation-immune and early left ventricular function in STEMI patients, and provides guidance and new ideas for the clinical application of PCSK9 inhibitors and the treatment of STEMI patients.

## Data Availability

The raw data supporting the conclusions of this article will be made available by the authors, without undue reservation.

## Glossary

ACEI: Angiotensin converting enzyme inhibitor
ACS: Acute coronary syndrome
APO A1: Apolipoprotein A-1
APO B: Apolipoprotein B
ARB: Angiotensin receptor blocker
ARNI: Angiotensin receptor neprilysin inhibitor
BMI: Body mass index
CABG: Coronary artery bypass grafting
CAG: Coronary angiography
CCL2: C-C motif ligand 2
CCR2: C-C chemokine receptor 2
CK: Creatine kinase
CK-MB: Creatine kinase-MB
Cr: Creatinine
DAPT: Dual antiplatelet therapy
DBP: Diastolic pressure
EDTA: Ethylenediaminetetraacetic acid
ELISA: Enzyme linked immunosorbent assay
eGFR: Estimated glomerular filtration rate
HDL-C: High-density lipoprotein cholesterol
HF: Heart failure
HR: Heart rate
hsCRP: High-sensitivity C-reactive protein
hs-cTnT: High sensitive-cardiac troponin t
IL: Interleukin
LAD: Left atrial diameter
LDL-C: Low-density lipoprotein cholesterol
Lp(a): Lipoprotein (a)
LV: Left ventricular
LVDd: Left ventricular end diastolic diameter
LVEF: Left ventricular ejection fraction
MACE: Major adverse cardiovascular events
MI: Myocardial infarction
NLR: Neutrophil-to-lymphocyte ratio
NLRP3: NOD-like receptor thermal protein domain associated protein 3
NT-proBNP: N terminal-pro-brain natriuretic peptide
PCI: Percutaneous coronary intervention
PCSK9: Proprotein convertase subtilisin/kexin type 9
PCSK9i: Proprotein convertase subtilisin/kexin type 9 inhibitors
RCT: Randomized controlled trial
SBP: Systolic blood pressure
SIRT3: NAD-dependent deacetylase sirtuin-3
STEMI: ST-elevation-myocardial infarction
TC: Total cholesterol
TGF-β: Transforming growth factor-β
TLR4: Toll-like receptor 4
TNF-α: Tumor necrosis factor-α
VLDL-C: Very low-density lipoprotein
