# Supplementary material for: Evolocumab early reduces IL-1β/IL-17A and improves left ventricular function in STEMI: an observational study in the real world
Source: Front Pharmacol. 2026 May 8;17:1800539. doi: 10.3389/fphar.2026.1800539 (PMC13194611; doi:10.3389/fphar.2026.1800539)
Supplement: Supplementary file 1 [file Table1.docx]

| **Table S1.** Comparison of lipids in patients with STEMI | | | | | | |
| --- | --- | --- | --- | --- | --- | --- |
| **Parameter** | **Group** | **Baseline** | **After 4 weeks** | **P** | **After 12 weeks** | **P** |
| LDL-C  (mmol/L) | Statin  Evolocumab  P | 3.41±0.66  3.58±0.55  0.933 | 1.99±0.70  0.90±0.49  0.179 | **<0.001**  **<0.001** | 1.97±0.57  0.93±0.49  **0.034** | **<0.001**  **<0.001** |
| nonHDL-C  (mmol/L) | Statin  Evolocumab  P | 3.81±0.71  4.03±0.65  0.521 | 2.38±0.73  1.24±0.58  0.220 | **<0.001**  **<0.001** | 2.29±0.65  1.25±00.63  **0.039** | **<0.001**  **<0.001** |
| APO B  (mmol/L) | Statin  Evolocumab  P | 1.08±0.17  1.16±0.17  0.453 | 0.80±0.24  0.56±0.25  0.509 | **<0.001**  **<0.001** | 0.85±0.22  0.58±0.24  **0.007** | **<0.001**  **<0.001** |
| Lp(a)  (nmol/L) | Statin  Evolocumab  P | 200.10(137.17,319.30)  255.62(108.31,404.00)  0.646 | 191.65(122.11,347.00)  202.77(86.38,512.00)  0.760 | 0.429  **<0.001** | 187.63(116.01,311.45)  174.44(102.91,398.10)  0.939 | 0.386  **<0.001** |

| **Table S2.** Comparison of inflammatory factors in patients with STEMI | | | | | | |
| --- | --- | --- | --- | --- | --- | --- |
| **Parameter** | **Group** | **Baseline** | **After 1 week** | **P** | **After 4 weeks** | **P** |
| IL-1β  (pg/ml) | Statin  Evolocumab  P | 16.64±2.51  16.95±2.64  0.338 | 15.06±1.92  14.83±2.15  0.352 | **<0.001**  **<0.001** | 12.48±1.51  11.96±1.49  **0.010** | **<0.001**  **<0.001** |
| IL-18  (pg/ml) | Statin  Evolocumab  P | 74.00±10.70  72.85±10.92  0.393 | 72.74±7.95  72.28±7.86  0.672 | 0.494  0.479 | 71.18±6.69  70.28±6.07  0.389 | **0.034**  0.123 |
| IL-17A  (pg/ml) | Statin  Evolocumab  P | 5.73±1.11  5.64±1.25  0.544 | 4.85±0.76  4.80±0.73  0.628 | **<0.001**  **<0.001** | 4.21±0.61  3.88±0.48  **<0.001** | **<0.001**  **<0.001** |
| CCR2  (ng/g) | Statin  Evolocumab  P | 1.66±0.37  1.64±0.34  0.691 | 1.25±0.37  1.27±0.34  0.530 | **<0.001**  **<0.001** | 0.92±0.19  0.90±0.16  0.420 | **<0.001**  **<0.001** |
| hsCRP  (mg/L) | Statin  Evolocumab  P | 5.09(1.57,10.86)  4.95(2.24,8.45)  0.742 | 5.16(1.85,10.45)  5.25(2.25,9.96)  0.640 | 0.066  0.133 | 1.68(0.71,3.45)  1.55(0.68,3.76)  0.829 | **<0.001**  **<0.001** |

STEMI, ST-segment elevation myocardial infarction. LDL-C, low-density lipoprotein cholesterol; nonHDL-C, non- high-density lipoprotein cholesterol; APO B, , apolipoprotein B; Lp(a), lipoprotein (a) . Data expressed as mean ± SD or median [interquartile range]. Differences were tested using the unpaired or paired t-test or the unpaired or paired Mann Whitney test as appropriate.

STEMI, ST-segment elevation myocardial infarction; IL-1β, interleukin-1β; IL-18, interleukin-18; IL-17A, interleukin-17A; CCR2, c-c motif chemokine receptor 2; hsCRP, hypersensitive C-reactive protein. Data expressed as mean ± SD or median [interquartile range]. Differences were tested using the unpaired or paired t-test or the unpaired or paired Mann Whitney test as appropriate.

| **Table S3. Comparison of changes in LV parameters in patients with STEMI grouped by LVEF** | | | | | |
| --- | --- | --- | --- | --- | --- |
| **Parameter** | **Group** | **After 1 week^#^/4 weeks*** | | **After 4 weeks^#^/12 weeks*** | |
|  |  | LVEF≥50% (n=133) | LVEF<50% (n=124) | LVEF≥50% (n=133) | LVEF<50% (n=124) |
| ΔIL-1β  (pg/ml) | Statin  Evolocumab  P | -6.96%±21.27%  -8.57%±22.98%  0.685 | -8.60%±17.39%  -11.07%±20.56%  0.455 | -26.21%±11.30%  -28.77%±12.91%  0.295 | -24.37%±10.49%  -29.87%±11.40%  **0.011** |
| ΔIL-18  (pg/ml) | Statin  Evolocumab  P | 0.03%±19.61%  -0.27%±18.97%  0.938 | 2.36%±19.70%  1.84%±16.06%  0.883 | -1.16%±18.93%  -6.09%±17.73%  0.258 | -2.05%±17.25%  3.57%±15.27%  0.130 |
| ΔIL-17A  (pg/ml) | Statin  Evolocumab  P | -9.81%±23.71%  -9.30%±24.39%  0.905 | -14.68%±20.31%  -11.75%±24.37%  0.452 | -24.19%±17.66%  -28.56%±15.34%  0.187 | -26.45%±14.12%  -31.67%±14.96%  **0.041** |
| ΔCCR2  (ng/g) | Statin  Evolocumab  P | -22.97%±29.71%  -21.89%±22.59%  0.814 | -17.73%±34.75%  -16.35%±28.96%  0.815 | -41.73%±19.69%  -42.47%±17.34%  0.890 | -41.96%±15.65%  -42.34%±15.20%  0.820 |
| ΔhsCRP  (mg/L) | Statin  Evolocumab  P | 4.61%(-58.18%,209.03%)  -1.84%(-49.63%,91.19%)  0.601 | -22.5%(-65.28%,128.02%)  -16.65%(-48.65%,87.88%)  0.701 | -51.44%(-85.72%,11.05%)  -70.42%(-86.59%,42.07%)  0.524 | -74.68%(-91.95%,-30.97%)  -73.50%(-88.28%,21.31%)  0.518 |
| ΔLAD  (mm) | Statin  Evolocumab  P | 0.63±3.28  -0.63±4.22  0.122 | 0.26±5.18  -0.11±3.66  0.698 | 0.19±3.62  -1.18±3.27  0.078 | 0.40±2.96  -0.71±3.17  **0.049** |
| ΔLVDd  (mm) | Statin  Evolocumab  P | 1.18±3.77  0.13±3.16  0.183 | -0.23±4.54  -0.97±3.70  0.427 | 0.48±1.00  -0.94±3.14  0.127 | -0.23±3.02  -1.62±3.21  **0.045** |
| ΔLVEF  (%) | Statin  Evolocumab  P | 4.73±6.71  3.13±5.49  0.196 | 5.62±6.39  8.06±8.90  0.129 | 2.39±7.22  4.20±5.94  0.183 | 7.40±8.07  11.36±7.19  **0.014** |

Data expressed as mean ± SD or median [interquartile range]. Differences were tested using unpaired t-test or the Mann Whitney test as appropriate. IL-1β, interleukin-1β; IL-18, interleukin-18; IL-17A, interleukin-17A; CCR2, c-c motif chemokine receptor 2; hsCRP, hypersensitive C-reactive protein. LAD, left atrial diameter; LVDd, left ventricular end diastolic diameter; LVEF, left ventricular ejection fraction.

**#** The differences between the inflammatory-immune factors after 1 week or 4 weeks and at baseline in 257 STEMI patients from the two study groups.

***** The differences between the parameters of LV function after 4 weeks or 12 weeks and at baseline in 257 STEMI patients from the two study groups.

| **Table S4.** Comparison of UCG parameters in patients with STEMI | | | | | | |
| --- | --- | --- | --- | --- | --- | --- |
| **Parameter** | **Group** | **Baseline** | **After 4 weeks** | **P** | **After 12 weeks** | **P** |
| LAD  (mm) | Statin  Evolocumab  P | 37.04±4.80  37.00±3.56  0.933 | 37.44±3.98  36.59±3.39  0.179 | 0.065  0.635 | 37.51±4.79  36.28±3.12  **0.034** | **0.012**  0.319 |
| LVDd  (mm) | Statin  Evolocumab  P | 51.14±5.45  50.75±4.04  0.521 | 51.38±3.05  50.44±3.45  0.220 | 0.069  0.411 | 51.18±2.72  49.45±3.55  **0.039** | 0.682  **0.026** |
| LVEF  (%) | Statin  Evolocumab  P | 49.17±7.04  49.82±6.74  0.453 | 54.66±8.16  55.41±7.78  0.509 | **<0.001**  **<0.001** | 54.29±9.21  57.36±6.34  **0.007** | **<0.001**  **<0.001** |

UCG, ultrasound cardiogram; STEMI, ST-segment elevation myocardial infarction. LAD, left atrial diameter; LVDd, left ventricular end diastolic diameter; LVEF, left ventricular ejection fraction. Data expressed as mean ± SD. Differences were tested using the unpaired or paired t-test as appropriate.

| **Table S5.** Comparison of changes in LV function parameters in patients with STEMI | | | |
| --- | --- | --- | --- |
| **Parameter** | **Group** | **After 4 weeks** | **After 12 weeks** |
| ΔLAD  (mm) | Statin  Evolocumab  P | 0.44±4.33  -0.40±3.97  0.179 | 0.29±3.70  -0.94±3.51  **0.034** |
| ΔLVDd  (mm) | Statin  Evolocumab  P | 0.38±4.23  -0.37±3.44  0.134 | 0.04±3.60  -1.27±3.21  **0.028** |
| ΔLVEF  (%) | Statin  Evolocumab  P | 5.19±6.53  5.38±7.62  0.852 | 5.07±8.05  7.53±7.44  **0.030** |

STEMI, ST-segment elevation myocardial infarction; LAD, left atrial diameter; LVDd, left ventricular end diastolic diameter; LVEF, left ventricular ejection fraction. Changes (Δ) were obtained by calculating the differences between the value of the parameter after 4 or 12 weeks and at baseline from the two study groups. Data expressed as mean ± SD. Differences were tested using the unpaired t-test.

| **Table S6.** Association between treatment with Evolocumab and inflammatory biomarkers | | | | | | | | | | |
| --- | --- | --- | --- | --- | --- | --- | --- | --- | --- | --- |
| Evoloc-umab | IL-1β | | IL-18 | | IL-17A | | CCR2 | | hsCRP | |
|  | Estimate β (95% CI) | *P* value | Estimate β (95% CI) | *P* value | Estimate β (95% CI) | *P* value | Estimate β (95% CI) | *P* value | Estimate β (95% CI) | *P* value |
| 0 | ref | - | ref | - | ref | - | ref | - | ref | - |
| 1 | -0.18  (-0.31, -0.04) | **0.012** | 0.16  (-0.56,-0.87) | 0.667 | -0.07  (-0.13,-0.01) | **0.030** | 0.00  (-0.02,0.02) | 0.701 | 1.03  (-0.08,2.15) | 0.070 |

CI: confidence interval ; IL-1β, interleukin-1β; IL-18, interleukin-18; IL-17A, interleukin-17A; CCR2, c-c motif chemokine receptor 2; hsCRP, hypersensitive C-reactive protein. The correlation and p value are estimated by the generalized estimation equation in 257 STEMI patients from the two study groups.

| **Table S7.** Association between treatment with Evolocumab and LV function parameters | | | | | | |
| --- | --- | --- | --- | --- | --- | --- |
| Evoloc-umab | LAD | | LVDd | | LVEF | |
|  | Estimate β  (95% CI) | *P* value | Estimate β  (95% CI) | *P* value | Estimate β  (95% CI) | *P* value |
| 0 | ref | - | ref | - | ref | - |
| 1 | -0.12(-0.22,-0.02) | **0.019** | -0.10(-0.19,0.00) | **0.042** | 0.18(0.01,0.34) | **0.036** |

CI: confidence interval ; LAD, left atrial diameter; LVDd, left ventricular end diastolic diameter; LVEF, left ventricular ejection fraction. The correlation and p value are estimated by the generalized estimation equation in 257 STEMI patients from the two study groups.

| **Table S8.** Association between treatment with β-blocker and ACEI/ARB/ARNI and inflammatory factors. | | | | | | | | |
| --- | --- | --- | --- | --- | --- | --- | --- | --- |
|  | IL-1β | | IL-18 | | IL-17A | | CCR2 | |
|  | Estimate β (95% CI) | | Estimate β (95% CI) | | Estimate β (95% CI) | | Estimate β (95% CI) | |
|  | Univariate | Multivariate | Univariate | Multivariate | Univariate | Multivariate | Univariate | Multivariate |
| β-blocker | -0.08  (-0.41,0.25) | -0.35  (-0.70,0.01) | -0.73  (-2.40,0.94) | -0.37  (-2.19,1.45) | -0.10  (-0.25,0.06) | 0.04  (-0.11,0.19) | -0.01  (-0.05,0.04) | 0.01  (-0.04,0.06) |
| ACEI/ARB/ARNI | 0.49*  (0.16,0.82) | 0.33  (-0.02,0.68) | -0.98  (2.79,0.84) | -0.67  (-2.65,1.32) | -0.01  (-0.16,0.15) | -0.09  (-0.24,0.06) | -0.04  (-0.08,0.01) | -0.05  (-0.09,0.01) |

CI: confidence interval ; IL-1β, interleukin-1β; IL-18, interleukin-18; IL-17A, interleukin-17A; CCR2, c-c motif chemokine receptor 2; ACEI, angiotensin converting enzyme inhibitors; ARB, angiotensin receptor blocker; ARNI, angiotensin receptor neprilysin inhibit; The correlation and p value are estimated by the generalized estimation equation in 257 STEMI patients from the two study groups. Multivariate analysis adjusted for baseline covariates: age, sex, smoking, drinking, medical history, and postoperative medications. **p* <0.05.
